# Assessing socioeconomic vulnerability to dengue fever in Cali, Colombia: statistical vs expert-based modeling

**DOI:** 10.1186/1476-072X-12-36

**Published:** 2013-08-14

**Authors:** Michael Hagenlocher, Eric Delmelle, Irene Casas, Stefan Kienberger

**Affiliations:** 1 Interfaculty Department of Geoinformatics – Z_GIS, University of Salzburg, Schillerstraße 30, Salzburg 5020, Austria; 2 Department of Geography and Earth Sciences, University of North Carolina at Charlotte, Charlotte, NC 28223, USA; 3 Department of Social Sciences, Louisiana Tech University, Ruston, LA 71272, USA

**Keywords:** Dengue fever, Vulnerability, Composite indicators, Expert-based vs. statistical modeling, GIS, Geovisualization, Vector-borne diseases, Colombia

## Abstract

**Background:**

As a result of changes in climatic conditions and greater resistance to insecticides, many regions across the globe, including Colombia, have been facing a resurgence of vector-borne diseases, and dengue fever in particular. Timely information on both (1) the spatial distribution of the disease, and (2) prevailing vulnerabilities of the population are needed to adequately plan targeted preventive intervention. We propose a methodology for the spatial assessment of current socioeconomic vulnerabilities to dengue fever in Cali, a tropical urban environment of Colombia.

**Methods:**

Based on a set of socioeconomic and demographic indicators derived from census data and ancillary geospatial datasets, we develop a spatial approach for both expert-based and purely statistical-based modeling of current vulnerability levels across 340 neighborhoods of the city using a Geographic Information System (GIS). The results of both approaches are comparatively evaluated by means of spatial statistics. A web-based approach is proposed to facilitate the visualization and the dissemination of the output vulnerability index to the community.

**Results:**

The statistical and the expert-based modeling approach exhibit a high concordance, globally, and spatially. The expert-based approach indicates a slightly higher vulnerability mean (0.53) and vulnerability median (0.56) across all neighborhoods, compared to the purely statistical approach (mean = 0.48; median = 0.49). Both approaches reveal that high values of vulnerability tend to cluster in the eastern, north-eastern, and western part of the city. These are poor neighborhoods with high percentages of young (i.e., < 15 years) and illiterate residents, as well as a high proportion of individuals being either unemployed or doing housework.

**Conclusions:**

Both modeling approaches reveal similar outputs, indicating that in the absence of local expertise, statistical approaches could be used, with caution. By decomposing identified vulnerability “hotspots” into their underlying factors, our approach provides valuable information on both (1) the location of neighborhoods, and (2) vulnerability factors that should be given priority in the context of targeted intervention strategies. The results support decision makers to allocate resources in a manner that may reduce existing susceptibilities and strengthen resilience, and thus help to reduce the burden of vector-borne diseases.

## Background

Severe outbreaks of vector-borne diseases (VBDs) and their expansion pose a serious challenge to vulnerable populations. Recent changes in climatic conditions, greater resistance to insecticides and new public health policies have changed the dynamics of VBDs, such as dengue fever or malaria [1-3]. Projected changes in climate conditions [4] along with other factors, such as population growth, urbanization, lack of sanitation, and ineffective mosquito control are expected to result in a geographical expansion of dengue fever in the coming decades [5]. While malaria is still the most significant communicable disease [2,6], dengue fever outbreaks have recently resurfaced [7,8]. Dengue fever is a vector-borne viral infection, transmitted among humans by the female *Aedes aegypti* mosquito [9]. It is prevalent in many tropical and sub-tropical regions across the globe [10-15]. Urban and suburban environments in those regions are particularly fragile due to rapid population movement (e.g. massive influx of migrants, causing unorganized urbanization) and the abundance of potential breeding sites.

In Colombia, South America, dengue fever reemerged in the 1970s after being eradicated in the 1950s and 1960s [16]. Ever since the disease has become endemic, presenting periodic outbreaks in 1991, 1994, 1998, 2001, 2006, and more recently in 2010. Most outbreaks have been of serotype 1 (DENV-1) and 2 (DENV-2) [17], however, in the last decade type 3 (DENV-3) and type 4 (DENV-4) have also been present [18]. Individuals infected with a particular serotype develop immunity to that type. When a serotype has not circulated for a while the population at risk of contracting the disease increases (in Colombia, this number currently equals 26 million individuals). These individuals are generally confined below 1,800 meters elevation, corresponding to nearly 80% of the total area of the country. Dengue fever in the city of Santiago de Cali (referred to as Cali from here on) has followed a similar temporal pattern to that of Colombia in general [18]. Based on the City’s Health Municipality, significant dengue outbreaks occurred in 1995 with 6,433 cases reported, in 2002 (n = 4,358), 2005 (n = 2,338) and 2010 (n = 9,600) [17-19]. In 2010, the total number of cases was the highest in the last 25 years.

Three critical areas warranting efforts for reducing the burden of dengue fever are (1) identifying factors responsible for its distribution, (2) conducting proactive programs to reduce existing health vulnerabilities, and (3) strengthening existing capacities for creating more resilient societies on all levels (i.e., from global to local). To be effective, these programs must be based on up-to-date and reliable information on existing vulnerabilities and capacities on site, which is the scope of our paper.

Vulnerability is a well-established concept within the disaster risk reduction and the climate change adaptation communities [20-23]. As it helps to identify intervention options for reducing susceptibilities and strengthening resilience to VBDs independent of current disease prevalence, the emerging concept of vulnerability assessment holds promises in public health. To date, some studies have been published on vulnerability to VBDs in general [3,24-26], and dengue in particular [27-31]. A handful of those have addressed vulnerability to dengue fever in Latin America [27,28]. De Mattos Almeida et al. [27] analyzed the link between different risk categories and socioeconomic, demographic and urban-infrastructure characteristics in an urban area of Brazil, while Martinez et al. [28] used Geographic Information Systems (GIS) for mapping vulnerability to dengue in the City of Havana, Cuba. However, these studies lack a clear conceptual vulnerability framework, thus making the comparability and reproducibility of their results difficult. Most research integrating geospatial analysis for monitoring the dynamics of dengue fever has focused on developing disease surveillance systems [32], assessing exposure [33], or measuring determinants for dengue infection [34], while vulnerability is not integrated. Strategies which solely focus on vector control, reducing exposure or treatment of the disease may provide noticeable health benefits, but could be less effective at reducing the total health burden in the long term than integrative approaches aimed at underlying causes of vulnerability [31,35]. This is also supported by Jones and Williams [36], who advocate for an integrated approach to infectious disease control.

The objective of this paper is to perform a spatial assessment and evaluation of relative levels of socioeconomic vulnerability between different neighborhoods in Cali, Colombia independent of dengue fever prevalence. In the wider context of dengue risk, the focus is on modeling vulnerability (as a component of risk), i.e. the predisposition of the population of Cali of being adversely affected by the disease.

Based on a holistic vulnerability framework (i.e., a deductive approach) we use both statistical and expert-based approaches for the analysis and aggregation of singular socioeconomic and demographic indicators, and compare the outcomes using geo-statistical methods.

## Materials and methods

### Study area

**Figure 1:**
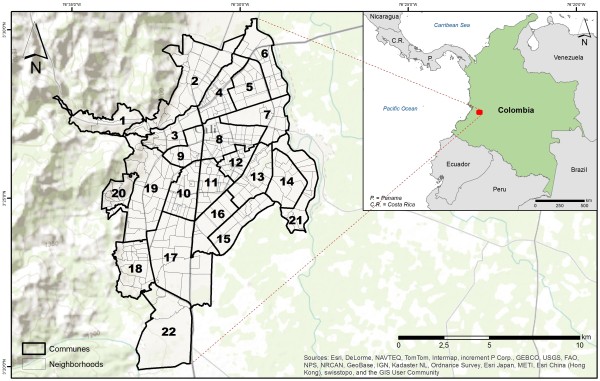
Base map showing the location of Cali, Colombia.

Cali’s core urban area is located west of the Cauca River with the Farallones Mountains acting as a natural barrier to the further expansion of the city. Cali generally experiences two rainy seasons from April to July and September to December. Located in an elevation of approximately 1,000 meters above sea level, the city’s average temperature is 26°C with an annual average precipitation of 1,000 mm covering most of the metropolitan area [37]. With an estimated population of 2.3 million, Cali is currently the third largest city in Colombia. The city is administratively organized in 22 communes, which are divided into 340 neighborhoods (Figure 1). Communes are neighborhood groupings based on homogeneous demographic and socioeconomic characteristics. Neighborhoods are stratified into six socioeconomic classes based on the type of housing, urban environment, and urban context, one being the lowest and six the highest. Peripheral neighborhoods are characterized by high-density, low-income population with unplanned urbanization, including squatter settlements along the river banks [38]. This has been exacerbated by the influx of migrants displaced by the armed conflict who can only afford to settle in areas where poor living conditions are present. Casas et al. [39] discuss the infrastructure of such regions, and its impact on health access. The poor infrastructure of these neighborhoods may result in open-air waste water channels, while several households make use of rainfall cisterns for drinking water [14]. Thus, ample sources of stagnant water (i.e., ideal mosquito-breeding habitats) are present around the city. Currently, health authorities in the city of Cali rely on a preventive dengue control strategy where they spray bacterial larvicides to stagnant water sources every two weeks.

### Vulnerability: conceptual framework

Our study makes use of the risk and vulnerability framework [40] which was developed in the European research project MOVE (Methods for the Improvement of Vulnerability Assessment in Europe). While the MOVE framework was elaborated in the context of natural hazard research and adaptation to climate change, it is modified in this paper to guide risk and vulnerability assessments in the VBD domain. The framework provides a conceptualization of the complex and multi-dimensional nature of vulnerability of a society and its population at different spatial and temporal scales. The MOVE framework characterizes vulnerability through three key factors, namely (1) exposure – reflecting the extent to which a unit of assessment falls within the geographical range of a hazard event, (2) susceptibility (SUS) – which describes the predisposition of elements at risk to suffer harm, and (3) lack of resilience (LoR), which is determined by limitations in terms of access to, and mobilization of the resources of a community or social-ecological system in responding to a particular hazard. As the MOVE framework intends to describe risk from a holistic perspective, different thematic dimensions characterize vulnerability: social, economic, environmental, physical, cultural and institutional [40]. In this paper, we define vulnerability as the predisposition of a system and its population of being adversely affected by the disease. Population vulnerability is determined by their degree of susceptibility, as well as the individual’s (lack of) resilience. In this conceptualization of vulnerability, the susceptibility (SUS) domain characterizes the predisposition of being negatively affected by an outbreak, whereas lack of resilience (LoR) includes (lacking) capacities to anticipate, cope or recover from the (burden of the) disease. In contradiction to the MOVE framework, we excluded the exposure component from the vulnerability analysis, as all neighborhoods have been affected by dengue outbreaks in recent years. We acknowledge exposure as part of the overall risk equation, where risk is defined by the location and magnitude of dengue occurrence, and the characteristics of exposed and vulnerable population groups. Since estimating dengue risk is not the focus of this paper, we assess socioeconomic vulnerability based on societal (i.e., the propensity for human well-being to be affected by disruption to individual and collective social systems and their characteristics) and economic (i.e., the propensity for loss of economic value from disruption of productive capacity) factors. By decomposing the complex, multi-dimensional phenomenon of vulnerability into its different components, the purpose of the framework is to serve as a guidance tool for the development of a representative set of indicators, suitable to represent socioeconomic vulnerability to dengue fever for the city of Cali, Colombia.

### Constructing a composite index of socioeconomic vulnerability

**Figure 2:**
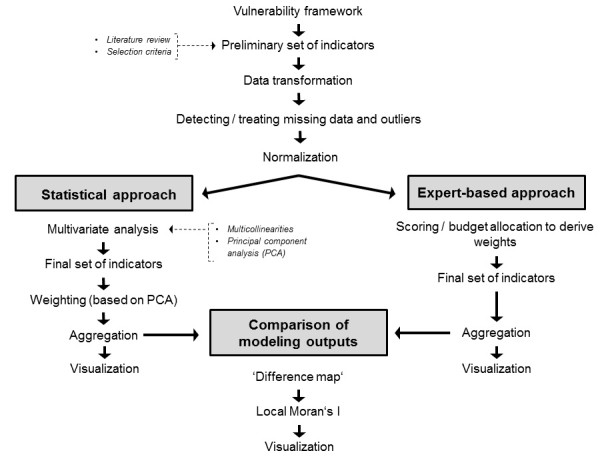
Study design and workflow.

In order to provide updated information on the multi-faceted nature of prevailing vulnerabilities to dengue fever in Cali, Colombia, a composite vulnerability index was developed. The index builds on a set of underlying socioeconomic and demographic indicators. A multi-step and iterative workflow (Figure 2) is adopted following OECD [41] guidelines. Relevant stages include: (1) definition of the conceptual framework, (2) identification of a representative set of indicators based on existing literature, (3) data transformation, (4) analysis and imputation of missing values, (5) normalization, (6) multivariate analysis and establishment of final indicator set, (7) weighting, (8) aggregation and (9) visualization, as detailed in Malczewski [42].

According to [41], there are multiple options for composite indicator construction. As indicated in Figure 2, we aim to compare the results derived by expert-based and statistical modeling approaches for the development of a socioeconomic vulnerability index. The main difference between both approaches is the way in which indicator weights are derived: While for the purely statistical approach multivariate analysis is used to derive indicator weights, the latter are assessed making use of traditional budget allocation for the expert-based approach (Figure 2). The following paragraphs provide an overview of the individual stages in constructing the composite vulnerability index.

Drawing on the conceptual vulnerability framework, within stage 2, a preliminary set of 23 socioeconomic indicators (Table 1) was identified based on a systematic review of literature and available datasets. The choice and selection of indicators and in particular socioeconomic factors, as outlined by Bates et al. [24,25], is a critical process in the overall method as it refers to evidence provided in scientific studies.

**Table 1:** **Preliminary list of susceptibility (SUS) and lack of resilience (LoR) indicators**^a^

**Domain**	**Indicator name**	**Domain**	**Indicator name**
SUS	Population density (km^2^)	LoR	Households without a phone (%)
SUS	Density of occupied households (km^2^)	LoR	People who cannot read or write (%)
SUS	Residents from age 0 to 4 (%)	LoR	People with no education (%)
SUS	Residents from age 5 to 14 (%)	LoR	People – secondary/higher education level (%)
SUS	Residents from age 15 to 29 (%)	LoR	Travel time to nearest hospital (min.)
SUS	Residents of age above 30 (%)	LoR	Distance to nearest hospital (m)
SUS	Black population (%)	LoR	Mean hospital density (km^2^)
SUS	White population (%)	LoR	Employed population (%)
SUS	People with disabilities (%)	LoR	Unemployed population (%)
SUS	Households without water access (%)	LoR	People doing housework (%)
SUS	Households without sewer system (%)	LoR	Retired people (%)
SUS	Building stratification (1–6)		

^a^Based on the outcomes of the literature survey and data availability (SUS = susceptibility; LoR = lack of resilience).

From literature population density, age groups, education levels, houses with different types of water access and the density of commercial areas have been identified as primary indicators in vulnerability assessments [27,28,31]. Despite dengue fever being regarded as a childhood disease [31], Guha-Sapir and Schimmer [43] observed that dengue hemorrhagic fever is also prevalent in older age groups. Variations of susceptibility levels in different age groups have also been observed in other studies [3,28,31,44]. Differences in race/ethnicity also have a marked impact on human susceptibility to dengue fever [43,45,46]. Sierra et al. [45], for example, observed that black individuals have a reduced risk for dengue fever compared to white/Caucasian individuals. Within the lack of resilience (LoR) domain, different levels of employment, and therefore access to financial resources, were identified as important coping and recovery indicators by Martinez et al. [28]. We included access to health facilities as an additional indicator, since adequate access to healthcare increases resilience (i.e., people’s capacity to cope and to recover) and may reduce dengue mortality [31]. We integrated building stratification – as a proxy for poverty levels and housing conditions – in the LoR domain, as for example poor housing quality was found to increase susceptibility to the disease [31]. Poor housing conditions facilitate movement of vectors between interior and exterior, but are also associated with a lack of other infrastructure, increasing the overall susceptibility to dengue [31].

During the selection process, standard criteria for indicator selection such as validity, sensitivity, reproducibility and scale [20,47] were accounted for. The indicators were associated with either the SUS or the LoR domain of vulnerability (Table 1).

The study builds on 2005 census data (i.e., the last census year) at the neighborhood level for the city of Cali which was made available by the municipality. It integrates ancillary geospatial datasets such as city roads, public transportation system network, and hospitals. The roads and public transportation network were obtained from various municipal agencies, while hospital addresses were acquired from the municipality and manually geocoded. This data was further processed using a GIS to calculate the average travel time between the centroid of each neighborhood and the closest hospital using public transportation [48] as well as the hospital density (in km^2^) in the city, by means of kernel density estimation. Finally, as vulnerability is a human-centered concept [49], all neighborhoods without permanent population (i.e., ‘residents’) were excluded from further analysis. This includes public parks, industrial areas, water treatment plants, military bases, cemeteries and academic institutions.

Within stage 3, raw data was transformed to achieve a better comparability of neighborhoods of different size and population or household counts [50]. For example, the absolute number of individuals who are not able to read or write was transformed into a relative measure. Following the same logic, areal density measures were computed in a GIS to transform the number of hospitals per neighborhood into a relative measure.

Descriptive statistics were used for each indicator within stage 4 to evaluate the degree of missing data and potential outliers. Following guidelines published by Groenefeld and Meeden [51], four indicators with skewness > 2 and kurtosis > 3.5 (i.e., hospital density; travel time to closest hospital; percentage of households without water; percentage of households without a phone) were highlighted as statistically ‘problematic’ with regard to potential outliers, and two indicators (i.e., building stratification; travel time to closest hospital) revealed a minimum percentage of missing data (< 2%). Missing values in the building stratification layer were imputed by integrating local expert-knowledge, while mean travel time to the closest hospital (in minutes) was imputed with the mean travel time of adjacent neighborhoods.

All datasets were standardized (stage 5) using linear min-max normalization (equation 1) and z-score standardization (equation 2), according to Malczewski [42].

(1) $$v_{i}^{\prime }=\frac{\left(v_{i}-v_{\min}\right)}{\left(v_{\max}-v_{\min}\right)}*\mathrm{sign}+0.5*\left(1-\mathrm{sign}\right)$$

Where *v*_*i*_ refers to the actual pixel value, *v*_*min*_ and *v*_*max*_ values derive from the original value range and *sign* (or polarity) indicates whether the indicator contributes positively or negatively to vulnerability. This results in normalized values (*v*_*i*_*’*) in the zero to one interval. Min-max normalization was chosen, as z-score produces negative indicator values and thus complicates the final aggregation of the indicators. Z-score standardization was selected as an additional technique as it produces output datasets with a mean of zero and a variance of one, which is useful for subsequent statistical analysis (e.g. principal component analysis).

(2) $$v_{i}^{\prime \prime }=\frac{\left(v_{i}-\bar{v}\right)}{\sigma }*\mathrm{sign}$$

with $\bar{v}$ corresponding to the mean and *σ* the standard deviation of the data. *V*_*i*_*”* is the resulting standardized value.

To detect and ultimately reduce existing multicollinearities in the data, the correlation coefficient (*r*), as well as variance inflation factors (VIF), were calculated within the SUS and LoR domain (stage 6). Based on thresholds published by the OECD [41], the following indicators (with *r* > 0.9 and/or VIF > 5) were excluded from further analysis: population density, proportion of white population, households without sewer system, people with no education, distance to closest hospital and proportion of population above 30 years.

For the statistical modeling approach, a principal component analysis (PCA) was conducted within each of the two domains to further test the robustness of the selected indicators (z-scores), and to derive indicator weights (as described below). Based on eigenvalues greater than 1.0 (Kaiser criterion), and a scree plot which shows a distinct break in the eigenvalues [41], the components which explain the majority of the variance among all neighborhoods were identified.

Weights were derived for the individual indicators (stage 7). Two different approaches were pursued to determine the weights to use (Figure 2). For the statistical approach, weights were calculated based on PCA and factor analysis [41]. For the expert-based approach, expert opinions were used to derive weights. Using a budget allocation approach, four local domain experts of differing backgrounds (epidemiologists and public health specialists from the Health Municipality of Cali) were asked to distribute a total of 100 points across the individual indicators within each of the two vulnerability domains (i.e., SUS, LoR).

The normalized indicators (min-max) were aggregated (stage 8) according to their respective domains (SUS, LoR):

(3) $$\mathrm{SUS}/\mathrm{LoR}=\frac{\sum_{i=1}^{n}w_{i}v_{i}^{\prime }}{n}$$

where *SUS/LoR* refers to either of the two vulnerability domains for a given neighborhood, *n* equals the number of indicators, *w*_*i*_ represents the weights for indicator *i* (either based on statistical or expert-based weighting; see Table 2) and *v*_*i*_^*’*^ is the normalized value (min-max) of indicator *i*.

**Table 2:** Final list of indicators, sign and weights (expert weights, statistical weights)

**Domain**	**Indicator name**	**Sign**^a^	**Expert weights**	**Statistical weights**	**Data source**
SUS_01	Density of occupied households (km^2^)	+	0.11	0.07	Census 2005
SUS_02	Residents from age 0 to 4 (%)	+	0.16	0.22	Census 2005
SUS_03	Residents from age 5 to 14 (%)	+	0.14	0.23	Census 2005
SUS_04	Residents from age 15 to 29 (%)	+	0.13	0.10	Census 2005
SUS_05	White population (%)	+	0.10	0.12	Census 2005
SUS_06	People with disabilities (%)	+	0.03	0.11	Census 2005
SUS_07	Building stratification (1–6)	-	0.14	0.15	Census 2005
SUS_08	Households without water access (%)	+	0.19	N/A	Census 2005
			*Sum =1*	*Sum = 1*	
LoR_01	Households without a phone (%)	+	0.03	0.17	Census 2005
LoR_02	People who cannot read or write (%)	+	0.09	0.16	Census 2005
LoR_03	Secondary/higher education (%)	-	0.13	N/A	Census 2005
LoR_04	Travel time to nearest hospital (min.)	+	0.12	0.08	Municipality
LoR_05	Mean hospital density (km^2^)	-	0.15	0.09	Municipality
LoR_06	Employed population (%)	-	0.11	0.12	Census 2005
LoR_07	Unemployed population (%)	+	0.18	0.11	Census 2005
LoR_08	People doing housework (%)	+	0.11	0.13	Census 2005
LoR_09	Retired people (%)	+	0.07	0.13	Census 2005
			*Sum = 1*	*Sum = 1*	

^a^Sign indicates if high indicator values increase (+) or decrease (−) vulnerability.

The aggregation of the two domains (i.e., SUS and LoR) into the final composite indicator of socioeconomic vulnerability was then performed using the equation below, while taking into account specific weights for the two domains as detailed below.

(4) $$\mathrm{VU}=\frac{\sum_{j=1}^{n}w_{j}X_{j}}{m}$$

In the equation, *VU* refers to the vulnerability index for a given neighborhood, *m* equals the number of domains, *w*_*j*_ represents the weights for domain *j* and *X*_*j*_ is the normalized value (min-max) of domain *j* (i.e., SUS, LoR). For both the statistical and the expert-based approach, a weight of 0.47 (eight SUS indicators divided by 17 total indicators) was assigned to the SUS domain, while the LoR domain was assigned a weight of 0.53 (nine LoR indicators divided by 17 total indicators). This was done to achieve a balanced structure between both domains within the composite vulnerability index.

To ease the interpretation of the modeling results, the final expert-based and statistical vulnerability index values were normalized within the zero to one range, where zero reflects a very low and one a very high socioeconomic vulnerability to dengue fever.

### Comparative assessment of modeling approaches

To assess differences in modeling outputs between the statistical and the expert-based approach, the difference (*Δ*) between both vulnerability indices was calculated (equation 5) and visualized in a GIS. Therefore, the normalized index values of the statistical approach (*VU*_*stat*_) were subtracted from the normalized values of the expert-based approach (*VU*_*exp*_):

(5) $$\Delta =VU_{\exp}-VU_{\mathrm{stat}}$$

To identify clusters of features with values similar in magnitude, a cluster analysis was performed using Anselin Local Moran’s I statistic [52] based on the difference (*Δ*) between both approaches.

## Results

### Vulnerability indicators

**Figure 3:**
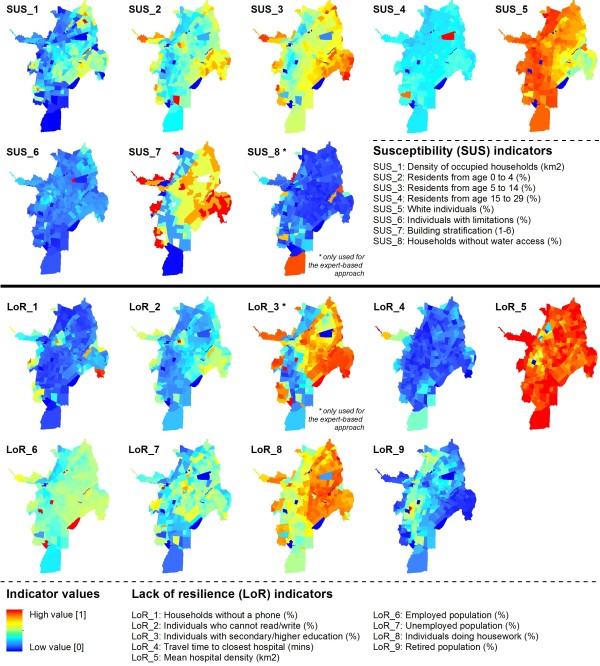
Final selection of susceptibility (SUS) and lack of resilience (LoR) indicators.

Following a multivariate statistical analysis (i.e., test for multicollinearities and PCA), 15 indicators were retained for statistical modeling. Two additional indicators (i.e., households without water access; secondary/higher education), that were excluded from the statistical modeling approach after multivariate analysis, were kept for the final construction of the expert-based composite vulnerability index as they were identified as relevant by the experts. Table 2 provides an overview of the final set of indicators and lists some of their properties, such as their sign, weights (including expert-based and statistical weights) and data source. Looking at the spatial distribution of the singular raw indicators (Figure 3) provides an initial idea how the city is characterized by the different socioeconomic variables.

### Socioeconomic vulnerability to dengue fever - comparing modeling approaches

**Figure 4:**
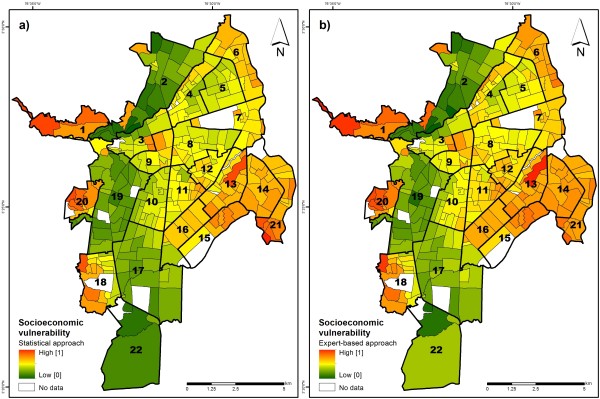
**Socioeconomic vulnerability to dengue fever in Cali, Colombia.** Figure 4a shows the results based on a statistical modeling approach, while Figure 4b shows the results based on an expert-based modeling approach.

Figure 4a illustrates the spatial variation in socioeconomic vulnerability to dengue fever based on statistical modeling. In contrast, Figure 4b shows the same results, for the expert-based modeling approach, where indicators and weights were selected based on expert opinion. In both maps, neighborhoods of high socioeconomic vulnerability are displayed in red, while neighborhoods with low values are displayed in green.

We conducted a Pearson correlation test among both expert-based and statistical vulnerability scores, indicating a strong positive relationship (*r* = 0.98). A comparison of descriptive statistics, however, has shown that the expert-based approach has a slightly higher mean (0.53) and median (0.56), compared to the purely statistical results (mean = 0.48; median = 0.49).

Both approaches revealed clusters of high levels of socioeconomic vulnerability in the eastern side of the city (comprising communes 13, 14, 15, 16 and 21), as well as the north-eastern side (commune 6), and western part of the city (comprising communes 1, 18 and 20). These are poor neighborhoods with high percentages of young (i.e., < 15 years) and illiterate residents, as well as a high proportion of individuals being unemployed or doing housework. Some neighborhoods bordering commune 3 and 9 also revealed higher vulnerability compared to other neighborhoods (Figure 4). These neighborhoods are characterized by high population density and lack of water infrastructure.

**Figure 5:**
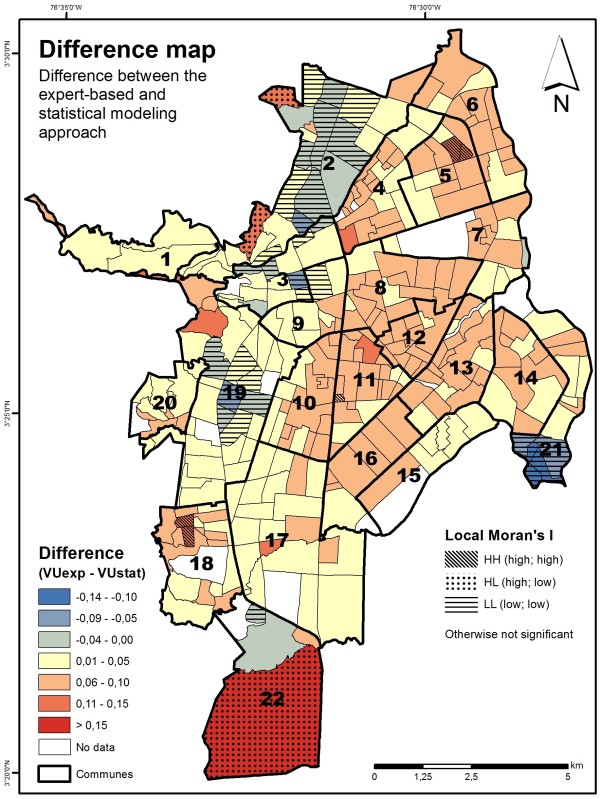
**Comparative analysis of statistical and expert-based modeling approaches.** Map showing the difference between both vulnerability indices (VU_exp_ – VU_stat_). The outcomes of a cluster analysis (Local Moran’s I) are displayed as an additional layer.

The difference between both modeling approaches is displayed in Figure 5.

The model outputs of both approaches have a high correlation, both globally, and spatially. There are, however, also distinct spatial discrepancies between both approaches. For example, within communes 2, 3, 19 or 21 the expert-based approach has revealed lower levels of vulnerability, while several communes in the eastern part (e.g. communes 8, 10, 11, 12, 13, 16) and southern part (commune 21) have revealed slightly higher vulnerability levels. This pattern can be partly explained by the fact that the expert-based assessment integrated two additional indicators (i.e., households without water access; secondary/higher education) which were removed for the statistical modeling. To highlight clusters of neighborhoods with significant differences, the outcomes of a local clustering method (Local Moran’s I) have been added as an additional layer (see Figure 5). HH (i.e., high; high) indicates that a neighborhood where the expert-based approach exhibits higher vulnerability values than the purely statistical approach is surrounded by other neighborhoods characterized by the same pattern. In contrast, LL (i.e., low; low) indicates that a neighborhood where the expert-based approach revealed lower vulnerability values than the purely statistical approach is surrounded by neighborhoods that show a similar pattern. HL (i.e., high; low) represents areas where the expert-based approach revealed higher vulnerability values that are surrounded by areas where the expert-based approach revealed lower vulnerability values than the statistical approach. Figure 5 also indicates that there is no significant local clustering in major parts of the city.

### Exploratory tools for visualizing multi-dimensional vulnerability

**Figure 6:**
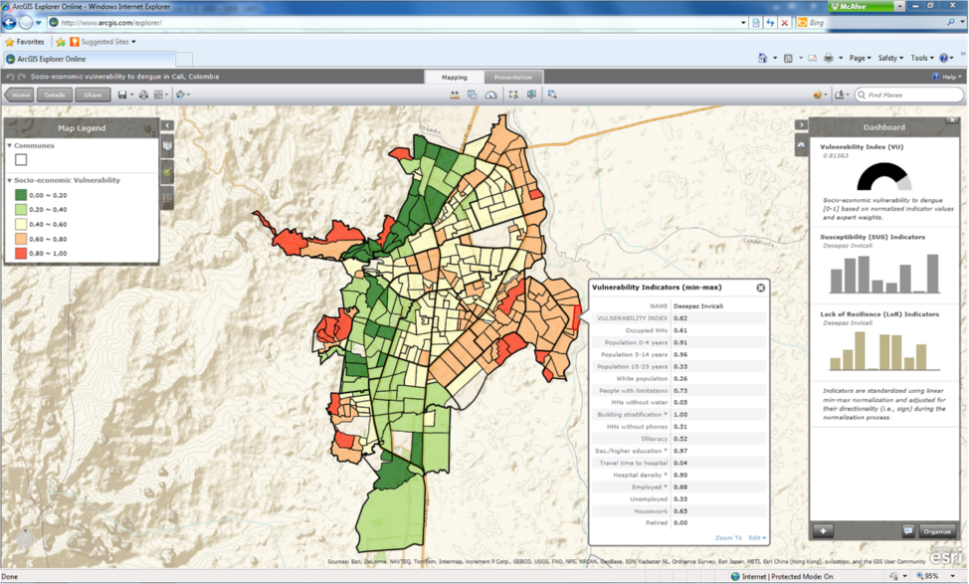
**Illustrative visualization of expert-based modeling results in ArcGIS explorer online.** Illustrative visualization of the results of the expert-based model (there was no particular reason why the expert-based model was selected; we could also display the results of the statistical model). This allows not only sharing results with a wider public, but also enables an assessment of the share of the underlying vulnerability indicators per neighborhood (see table and bar-charts in the figure).

As part of any vulnerability study, an interaction with the community and decision makers is highly recommended. In addition to traditional maps, the outcomes of the analyses were incorporated within an online visualization tool. Current web-based visualization tools offer tremendous capabilities for publishing and disseminating geospatial information to a world-wide audience [53]. We used ArcGIS Explorer Online as an advanced Web-GIS portal to help organize, analyze, and illustratively visualize the results of our study (Figure 6). Such Web-GIS portals provide a collaborative platform for users to explore vulnerabilities towards infectious diseases in general and dengue fever in particular. This dissemination platform is particularly attractive to decision makers who must allocate prevention resources: for example, when selecting a particular neighborhood, a decision maker may retrieve valuable information on the underlying vulnerability indicators, and their relationship to the vulnerability for the entire neighborhood. A nice attribute of this dissemination platform is that it can integrate additional visual outputs such as pie-charts, half-donuts, or bar charts.

## Discussion

Reducing the burden of vector-borne diseases without vaccines, such as dengue, requires integrated approaches that take into account both vector or pathogen exposure as well as human susceptibility to the disease [31]. Our study aimed at assessing prevailing socioeconomic vulnerabilities to dengue fever independent of the spatial distribution of the hazard (i.e., the disease) based on purely statistical and expert-based approaches taking into account a large set of representative census data and ancillary geospatial datasets; an approach which has not been pursued so far. The assessment was based on a holistic, integrative, yet decomposable, conceptual vulnerability framework. It served as a guidance tool for the establishment of a representative system of indicators, and thus enabled the construction of a composite vulnerability index based on appropriate indicators.

We identified vulnerability hotspots (i.e., clusters of neighborhoods with high levels of vulnerability to dengue fever) across the 340 neighborhoods in Cali, Colombia. By decomposing these hotspots into their underlying socioeconomic vulnerability factors (Figure 6), our approach not only provides information on the neighborhoods where intervention options are most warranted, but also indicates which factors need to be given priority to effectively reduce existing susceptibilities and increase resilience to the disease.

**Figure 7:**
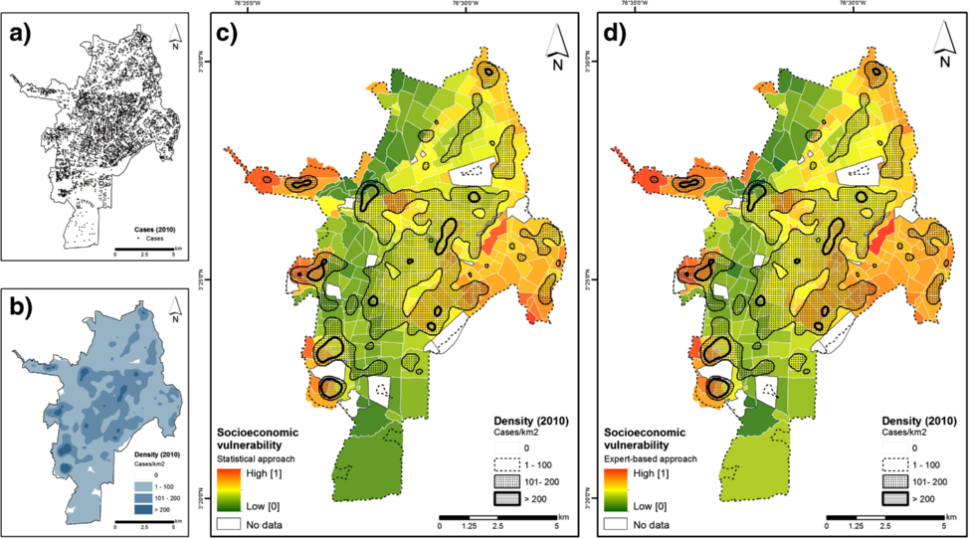
**Comparison of vulnerability at neighborhood level and dengue cases.** Figure 7a shows the spatial distribution of dengue cases (January-December, 2010). A density map (i.e., cases per km^2^) is shown in Figure 7b. The dengue density layer is displayed on top of the two vulnerability maps (Figures 7c, 7d) to enable a visual comparison of vulnerability at the neighborhood level and the spatial distribution of dengue cases.

The framework proposed in this paper also comes along with specific challenges. First, the modeling approach is not spatially explicit as it builds on indicators which were reported at the neighborhood level, thus neglecting the ‘true’ spatial distribution of existing vulnerabilities within the study area. It also results in ‘artificial boundaries’ that may further lead to aggregation problems as described by Openshaw [54]. Providing a more ‘realistic’ picture of existing vulnerabilities calls for spatially explicit modeling approaches, which enable a delineation of homogeneous units of vulnerability [55], independent of a-priori defined ‘artificial’ boundaries. Secondly, additional indicators such as extent and coverage of media campaigns or funding for dengue control programs [31] were not available and thus not integrated into the vulnerability analysis. Third, our approach is not temporally explicit: we considered the census and geospatial datasets at a particular point in time to construct a vulnerability index. Frequent updates and continuous monitoring of prevailing vulnerabilities are particularly needed in rapidly changing urban environments. Finally, one key challenge which warrants further research is the scientific validation of the modeling results. As vulnerability cannot be measured in real world, its validation remains both a hot topic as well as a scientific challenge [20,31,55]. However, when integrating the results of the vulnerability assessment in a dengue risk framework, the availability of incidence data (i.e., the manifestation of risk) could help to validate the resulting risk maps. Despite constraints associated with the validation of vulnerability assessments [20,31,55], a kernel density estimator was used to create a surface showing the density of dengue cases (in km^2^) for 2010 (January-December). Both, the spatial distribution of cases and the resulting dengue fever intensity layer, were mapped and the latter was visualized on top of the two vulnerability maps (Figure 7) to enable a visual comparison of vulnerable neighborhoods and areas at risk of contracting the disease (reflected by the dengue intensity surface).

Figure 7 shows that very high densities of dengue cases (i.e., > 200 cases/km^2^) tend to concentrate in the western, highly vulnerable fringes of the city, thus placing these areas at high risk. High densities (i.e., 101–200 cases/km^2^) are prevalent in the central and eastern part of the city (areas of medium socioeconomic vulnerability), while lower densities (i.e., 1–100 cases/km^2^) are distributed all over the city, also affecting areas of low socioeconomic vulnerability (Figure 7).

To increase user confidence in the applicability of the approach, future work will assess the sensitivity of the approach as well as the validity of the results by further investigating the spatial and statistical relationship between both the modeling results and the individual vulnerability indicators and dengue fever prevalence in the study area.

Findings from this research are particularly salient for public health authorities. First, for the planning of preventive control strategies, for example educational campaigns can be organized with participation from the community, which are likely to increase awareness and proper practices towards the virus. Focusing more resources on particularly vulnerable areas of the city can aid in changing people’s awareness and modify people’s behavior and attitude towards dengue fever (e.g., use of insecticide treated nets, etc.). It can also assist in planning preventive spraying of bacterial larvicides to permanent stagnant water sources. Second, it can facilitate timely response strategies during an outbreak by pointing towards areas that are more vulnerable to be controlled first, with the objective of minimizing the impact of the virus.

## Conclusions

Statistical and expert-based approaches were utilized for the modeling of prevailing vulnerabilities to dengue in the urban tropical environment of Cali, Colombia independent of the current distribution of the disease. Using neighborhoods as the spatial reporting unit, we integrated various socioeconomic and demographic indicators derived from census data and ancillary datasets into a final composite vulnerability index. A conceptual framework was used as a guidance tool for the development of a representative set of vulnerability indicators, thus enabling the reproducibility and comparability of modeling results. The methods presented in this paper make an important contribution as a decision support tool for reducing existing vulnerabilities and strengthening or building up resilience to vector-borne diseases in general, and dengue in particular. The results of our analysis also provide relevant information for decision makers in Cali, Colombia, as they not only help prioritizing intervention areas (i.e., vulnerability hotspots), but also indicate which factors need to be addressed foremost in the context of targeted intervention measures.

## Competing interests

The authors declare that they have no competing interests.

## Authors’ contributions

MH and SK were responsible for the conceptualization and design of the study. IC and ED were responsible for data collection and pre-processing. MH was responsible for data analysis, interpretation of results, and for drafting the manuscript (incl. the figures). ED, IC and SK provided substantial input to the manuscript and the interpretation of modeling results. All authors read and approved the final manuscript.
